# Long‐Term Neurodevelopmental Outcome Following Three Randomized Controlled Trials of Choline in Fetal Alcohol Spectrum Disorder (FASD)

**DOI:** 10.1111/acer.70394

**Published:** 2026-10-12

**Authors:** Jeffrey R. Wozniak, Judith K. Eckerle, Blake A. Gimbel, Abigail M. Ernst, Mary E. Anthony, Kent A. Tuominen, Erik de Water, Alexandra L. Doughty, Steven H. Zeisel, Michael K. Georgieff

**Affiliations:** ^1^ Department of Psychiatry & Behavioral Sciences University of Minnesota Minneapolis Minnesota USA; ^2^ Department of Pediatrics University of Minnesota Minneapolis Minnesota USA; ^3^ Department of Pediatric Psychology & Neuropsychology Nationwide Children's Hospital Columbus Ohio USA; ^4^ Department of Pediatrics The Ohio State University College of Medicine Columbus Ohio USA; ^5^ Great Lakes Neurobehavioral Center Eagan Minnesota USA; ^6^ University of North Carolina at Chapel Hill Chapel Hill North Carolina USA

**Keywords:** choline, cognition, fetal alcohol spectrum disorders, longitudinal studies

## Abstract

**Background:**

Fetal alcohol spectrum disorder (FASD) represents a range of neurodevelopmental outcomes from prenatal alcohol exposure for which there are few interventions. Preclinical studies have identified choline as a potential treatment for cognitive deficits in FASD. We previously conducted three randomized controlled trials (RCTs) in children under age 6 and reported on memory benefits following all three trials. Two RCTs compared a fixed 500 mg daily dose to placebo, and the third RCT tested a weight‐adjusted choline dose. Here, we report on a longitudinal follow‐up 5 years post‐study completion.

**Methods:**

The RCTs included 2.5‐ to 5.9‐year‐old children with FASD who received choline (*n* = 53) or placebo (*n* = 51). Participants described here (*n* = 28 choline; *n* = 30 placebo) were 9.1 years old on average at follow‐up. Of these, 9% had fetal alcohol syndrome (FAS), 43% partial FAS (PFAS), and 48% alcohol‐related neurodevelopmental disorder (ARND). Parents completed a diagnostic interview and behavior checklists while participants completed assessments of general cognitive ability, executive functioning, and memory.

**Results:**

Participants who received choline in the initial RCT showed a medium effect size performance advantage (9.1% better) for non‐verbal IQ and a medium effect size advantage (11.3% better) for working memory over placebo. Age at enrollment did not explain significant variance in outcome but choline dose was significantly associated with non‐verbal IQ and working memory (higher dose contributed to better performance at follow‐up). High rates of anxiety disorders and impulse‐control disorders were observed. While there were no statistically significant differences in mental health diagnoses for choline versus placebo, there were some differences that neared significance.

**Conclusions:**

Very few RCTs have been conducted in FASD, and interventions targeting cognitive functioning are needed. The data reported here suggest that choline supplementation has long‐lasting neurodevelopmental benefits, especially in non‐verbal IQ and working memory for individuals with FASD.

## Graphical abstract

This manuscript describes a 5‐year longitudinal follow‐up study of children with Fetal Alcohol Spectrum Disorders (FASD) who took part in one of three randomized controlled trials of choline supplementation when they were 2.5–5.9 years old. Results demonstrate that early choline supplementation improves long‐term non‐verbal IQ and working memory and that choline dose is associated with the level of improvement.

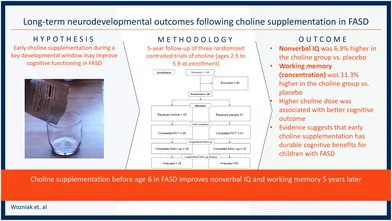

**Abbreviations**
ARND: Alcohol‐related neurodevelopmental disorder
CNS: Central nervous system
FAS: Fetal alcohol syndrome
FASD: Fetal alcohol spectrum disorders
PAE: Prenatal alcohol exposure
PFAS: Partial FAS
RCT: Randomized controlled trial

## 1 Introduction

Prenatal alcohol exposure (PAE) can result in a range of neurodevelopmental abnormalities that are diagnostically classified as fetal alcohol spectrum disorders (FASD). FASD is an umbrella term covering several diagnoses including fetal alcohol syndrome (FAS), Partial FAS (PFAS), and alcohol‐related neurodevelopmental disorder (ARND). The spectrum includes a range of growth deficits, dysmorphic facial features, brain anomalies, cognitive deficits, and behavioral challenges (Hoyme et al. 2016). Compared to typically developing children, global cognitive impairment occurs more frequently in FASD as do deficits in individual domains of functioning including attention, memory, executive functioning, visual‐perception, and motor skills (Panczakiewicz et al. 2016; Wozniak et al. 2019). These deficits can lead to life‐long challenges for affected individuals (Moore and Riley 2015).

FASDs represent a significant burden to public health in that they impact 1.0%–5.0% of the population globally (Lange et al. 2017; May et al. 2018). Furthermore, reports show that alcohol consumption rates around the world are rising, increasing the risk for alcohol‐exposed pregnancies (Castaldelli‐Maia et al. 2021). However, FASD continues to be underrecognized and underdiagnosed (Chasnoff et al. 2015) and support for the neurodevelopmental differences faced by individuals with FASD is very limited. At present, very few treatments for FASD have been developed and rigorously tested (Flannigan et al. 2020).

Choline is an essential nutrient that has been investigated in animal models and, minimally, in humans as a potential treatment for cognitive impairment following PAE (Ernst et al. 2022; Wozniak et al. 2020). Studies in animal models of PAE have found that choline supplementation directly impacts development of the brain and eye (Sawant et al. 2019) as well as motor skill development (Idrus et al. 2017). Animal models have also shown that choline supplementation has positive effects on spatial learning and hippocampal‐dependent learning and memory (Thomas et al. 2007; Waddell and Mooney 2017). Notably, studies have shown benefits from choline supplementation in the context of both typical and atypical development (Zeisel and Niculescu 2006). Choline is a precursor to the phospholipids required for axonal growth and repair, cell membrane formation, and myelination (Bekdash 2019; Zeisel 2011). Phospholipid formation and myelination are processes critical to the development and maintenance of white matter, which is responsible for rapid and efficient communication in the brain. Choline is also a precursor to the neurotransmitter acetylcholine, which is key to memory function and is especially prominent in the hippocampus (Cheng et al. 2008; Conlay and Zeisel 1982; Meck and Williams 1997). In the hippocampus, choline contributes to increased dendritic arborization in CA1, larger cells, and functional changes (Li et al. 2004; Pyapali et al. 1998). Choline also affects the hippocampal cholinergic system and alters development, structure, and function in regions essential for both memory and executive function, including methylation in the hippocampus and prefrontal cortex (Albright, Friedrich, et al. 1999; Albright, Tsai, et al. 1999; Zeisel 2006; Zeisel and Niculescu 2006). Glenn et al. (2008) found that prenatal choline supplementation in rats led to less age‐related cognitive decline along with increased proliferation of hippocampal cells, increased levels of vascular endothelial growth factor, and neurotrophin‐3 in the brains of the rats that received choline. Finally, choline is a methyl donor, which facilitates the regulation of DNA methylation and, in turn, influences gene expression during important developmental stages (Akison et al. 2018; Sarkar et al. 2019). The benefits seen from choline supplementation in children are likely due to a combination of these processes in brain development. Importantly, when choline is provided at early stages of brain development, the beneficial effects are believed to be long‐lasting and possibly permanent (Meck and Williams 2003; Zeisel and Niculescu 2006).

Few human intervention studies have been conducted testing the efficacy of choline supplementation (for comprehensive reviews, see (Akison et al. 2018; Ernst et al. 2022)). Prenatal choline supplementation (given to the mother during pregnancy) may have advantages over postnatal supplementation (given to the child after birth) due to the potential for positively impacting neurodevelopment during very early critical developmental windows (Akison et al. 2018). Studies of prenatal choline supplementation have suggested beneficial effects on cognitive functioning, regional brain volumes, and behavioral outcomes in infants with PAE (Coles et al. 2015; Jacobson et al. 2018; Warton et al. 2021). One study (Kable et al. 2015) examined the effect of choline on infant neurophysiology and memory encoding in pregnant women with moderate/heavy prenatal alcohol use. Participants, who were randomized to control, multivitamin only, or multivitamin plus choline (750 mg daily), were tested for cardiac orienting response (a habituation/dishabituation paradigm) at 12 months. Results indicated a benefit for multivitamin supplementation (with or without choline) on birth outcomes (longer gestation, increased birth weight, length, and head circumference), as well as a positive correlation between multivitamin plus choline supplementation and infant memory. Multivitamin supplementation (with or without choline) was also associated with higher cognitive functioning at 6 months (Coles et al. 2015).

Jacobson et al. (2018) conducted a randomized, double‐blind, placebo‐controlled clinical trial (RCT) of prenatal choline supplementation in South Africa and found that mothers who consumed alcohol and were then treated with choline had infants with superior eyeblink conditioning (better associative learning) at 6.5 months of age compared to placebo. Although newborns in both groups were small at birth, infants of choline‐treated mothers showed greater “catch‐up” in weight and head circumference at 6.5 and 12 months. At 12 months, infants in the choline supplementation group showed higher novelty preference scores (a measure of visual recognition memory) compared to those in the placebo group. In a follow‐up neuroimaging study in this cohort, the investigators showed that infants in the maternal choline group had normalized volumes relative to the placebo group in 6 of 12 brain regions including the bilateral thalamus and caudate, right putamen, and corpus callosum (Warton et al. 2021). In addition, increased volume in the right putamen and corpus callosum partially mediated the relationship between treatment group and recognition memory performance found in the original cohort at 12 months of age.

Our research team at the University of Minnesota began testing postnatal choline supplementation as a potential treatment for FASD in 2013 with a small pilot RCT (target enrollment *n* = 20; achieved enrollment *n* = 20) (Wozniak et al. 2013). This initial study, which was designed to have 80% power to detect a clinically meaningful Cohen's *d* effect size of 0.43 or greater and was designed to be eventually combined with RCT2, demonstrated the feasibility, safety, and tolerability of choline supplementation in 2.5‐ to 5.9‐year‐old children with FASD. We conducted a second RCT in 2015 (target enrollment *n* = 40; achieved enrollment *n* = 40), powered based on the observed effect size of *d* = 0.66 from RCT1, and we used the combined data from the two RCTs to evaluate the efficacy of the 500 mg per day choline dose for 9 months in children with FASD across the same age range (Wozniak et al. 2015). The target of these first two RCTs was cognitive functioning. Choline supplementation resulted in greater improvement in a hippocampal‐dependent measure of memory functioning than placebo. Results also suggested that children supplemented at a younger age (2.5–3.9 years) demonstrated greater memory performance benefits than those supplemented at an older age (4.0–5.9 years). The combined cohort completed follow‐up observational studies at 4 years and 7 years on average post‐RCT completion. Results indicated sustained cognitive benefits as well as significant group differences in brain white matter microstructure (increased fiber organization in the splenium of the corpus callosum) for the participants who had previously received choline (Gimbel et al. 2022; Wozniak et al. 2020). The first two RCTs combined found specific improvements in sequential memory (Wozniak et al. 2013, 2015) post hoc analyses suggested that the highest daily doses of choline per kilogram of body weight were associated with less improvement in memory performance at 9 months and higher occurrence of fishy body odor. The odor is the result of excess choline reaching the gut where bacteria then produce trimethylamine which is then excreted in sweat and urine. Because 500 mg appeared to be excessive for the smallest participants, we designed the third RCT (target enrollment *n* = 60; achieved enrollment *n* = 44) to use a weight‐adjusted choline dose (19 mg per kg of body weight). This study was truncated due to the COVID‐19 pandemic. As such, the combined data from all three RCTs were reported together (Wozniak et al. 2025). Those data revealed that, at study completion (9 months), choline supplementation resulted in improved performance on a hippocampus‐dependent memory task. The data also showed a dose–response relationship, with higher choline doses contributing to greater memory improvement. The combined dataset did not show an age‐moderated effect for choline.

Here, we present longitudinal follow‐up data for the combined cohort of child participants from all three RCTs who were treated with choline or placebo for 9 months starting between ages 2.5–5.9 years. Five years have passed between study completion and return for this follow‐up, allowing for an examination of the durability of the initial choline effect on cognition and behavior. These data provide valuable insights into the potential effects of modifying neurodevelopmental trajectories at an early stage on long‐term functioning for individuals with FASD.

## 2 Materials and Methods

### 2.1 Participants

All participants in the current longitudinal follow‐up study were initially enrolled in one of three RCTs of choline supplementation in FASD, the details of which have been previously published (Wozniak et al. 2013, 2015, 2025). Parents completed a comprehensive informed consent process. All procedures were approved by the University of Minnesota's Institutional Review Board prior to study initiation. Additional oversight of the trials was provided by a University clinical trial monitoring program and three independent Data Safety Monitoring Boards that included pediatricians and child psychologists. Choline was administered under the Federal Drug Administration Investigational New Drug (IND) application #107085. The trials were registered on ClinicalTrials.gov as NCT01149538 (6/21/2010)—[RCT1], NCT02735473 (4/2/2016)—[RCT2], and NCT05108974 (10/26/2021)—[RCT3] prior to enrollment of the first participant in each respective study. Participants were re‐consented as part of the current longitudinal follow‐up study. The clinical trial protocols and statistical plan are available by request from the corresponding author.

Children, ages 2.5–5.9 years at the time of enrollment, were recruited from the University of Minnesota's FASD diagnostic clinic as well as community FASD clinics and support groups for families raising children with FASD. All participants were living with adoptive families or in other permanent placements such as kinship care (none were in foster care). Exclusion criteria were: other neurodevelopmental disorders at the time of enrollment (ex. autism spectrum disorder, down syndrome, etc.) except attention‐deficit/hyperactivity disorder (ADHD), neurological conditions (ex. epilepsy, traumatic brain injury, etc.), medical conditions impacting the brain, or known history of very low birthweight (less than 1500 g). ADHD was not exclusionary because it is very common in FASD (O'Connor et al. 2002). Autism, which can co‐occur with FASD but generally represents a different developmental trajectory in this age range, was excluded in order to reduce variability in behavioral and cognitive presentation so as to maximize the opportunity to measure changes in developmental trajectory due to choline. Polysubstance use is also very common in FASD (Popova et al. 2024), and we have previously reported that treatment groups across each of the three RCTs did not differ on polysubstance exposure (Wozniak et al. 2025). Therefore, we did not exclude participants for comorbid drug exposure.

Across the three RCTs, 149 children were screened and 109 children who were eligible and interested were ultimately randomized (Figure 1). Of these, 104 participants were enrolled and received the allocated intervention. Across the three RCTs, 20 participants discontinued prior to the final trial visit (see Figure 1 for reasons), resulting in 84 participants who completed one of the three trials. Attempts were made to re‐contact all 84 participants who completed one of the RCTs. Of these, 58 ultimately participated in the current longitudinal follow‐up study. A comparison of participants who completed the longitudinal follow‐up versus those who did not is included in the Results section.

**FIGURE 1:**
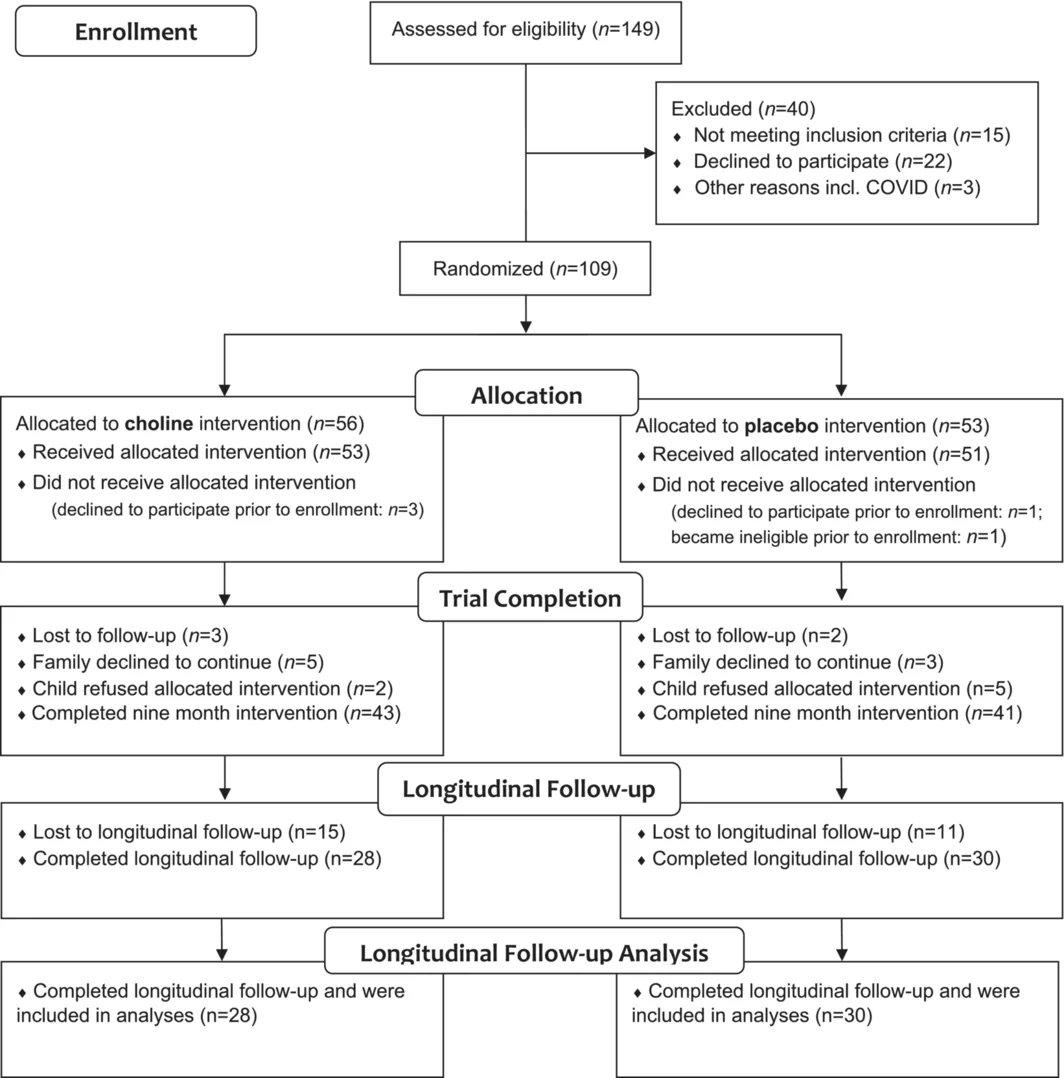
CONSORT flow diagram covering participants from all three randomized controlled trials.

### 2.2 Procedures

#### 2.2.1 Summary of Initial Trial Procedures

Details of the three parallel group superiority RCTs are contained in prior publications (Wozniak et al. 2013, 2015, 2025). Briefly, participants were randomly assigned (1:1 allocation) to choline versus placebo for 9 months; randomization tables were prepared ahead of the study by a blinded statistician. The Investigational Drug Services Pharmacy at the University of Minnesota supplied the study drug to participants in coded packets. Investigators and participants were blinded to the treatment group. All three RCTs used a parent‐administered supplement of choline or placebo given once daily over a 9‐month span. The treatment duration was chosen to allow for sufficient developmental change that would be detectable by the outcome instruments and was incorporated into all three RCTs to facilitate data pooling across the studies. Participants in RCT1 and RCT2 received 500 mg daily of choline (1.25 g choline bitartrate) or a placebo. RCT3 participants received a weight‐adjusted daily dose equal to 19 mg per kg of body weight or placebo; daily choline doses ranged from 290 to 500 mg. Parents combined the fruit‐flavored drink mix containing choline or placebo with water and provided the drink to the child each day. Adherence was high (78%–88% across studies) and has been described in detail elsewhere (Wozniak et al. 2025).

At baseline, participants completed cognitive testing prior to receiving choline or placebo. Participants in RCT1 (Wozniak et al. 2013) and RCT2 (Wozniak et al. 2015) were administered the Mullen Scales of Early Learning (Mullen 1995) as a measure of global cognitive functioning, while participants in RCT3 were administered the Stanford‐Binet Intelligence Scale, Fifth Edition (SB‐5) (Roid 2003). Although the Mullen and SB‐5 are not entirely equivalent batteries, both assess a range of cognitive functions including language, visual/non‐verbal processing, and motor skills, and both provide an index of global cognitive functioning. In addition, parents completed the Child Behavior Checklist (CBCL) (Achenbach and Rescorla 2000) at each of the three in‐person visits. At the 9‐month final RCT visit, participants were re‐administered the Mullen (RCT1 and RCT2) or SB‐5 (RCT3). Between baseline and study completion, eight telephone visits were completed to track compliance and collect adverse event information (starting 2 weeks post‐baseline and then monthly thereafter).

#### 2.2.2 Longitudinal Follow‐Up Procedures

Participants from RCT1 and RCT2 were re‐contacted and seen for a single follow‐up visit between December 2015 and November 2017. Results were described in a previous publication (Wozniak et al. 2020). Participants from RCT3 were re‐contacted and seen for a single follow‐up visit between October 2022 and March 2025. The current study includes combined longitudinal follow‐up data from participants in all three RCTs.

### 2.3 Measures

At the longitudinal follow‐up visit, all participants were administered the SB‐5 (Roid 2003), the *Memory for Names*, *Memory for Faces*, and *Narrative Memory* subtests from the Developmental Neuropsychological Assessment—2nd Edition (NEPSY‐II) (Korkman et al. 2007), and the Trailmaking and *Color‐Word Interference* subset from the Delis Kaplan Executive Function System (D‐KEFS) (Delis et al. 2001). The SB‐5 yields three overarching measures (*Full‐Scale IQ or FSIQ*, *Verbal IQ or VIQ*, and *Non‐verbal IQ or NVIQ*) in addition to five index scores: *Fluid Reasoning, Knowledge, Quantitative Reasoning, Visual–spatial Ability*, and *Working Memory*. SB‐5 scores have a mean of 100 and a standard deviation of 15, with higher scores indicating better performance. *Memory for Names* required participants to view drawings of faces and memorize the associated names over a series of trials with feedback. After a 30‐min delay, a recall trial tested long‐term memory for the faces. *Memory for Faces* required the child to view black and white photographs of faces and recall them in the presence of distractors, including a recall trial after a 20‐min delay. Narrative memory required the child to listen to and immediately freely recall a short story. Subsequently, a recognition trial was administered. All NEPSY‐II standardized scores have a mean of 10 and a standard deviation of 3, with higher scores representing better performance. *D‐KEFS Trail Making*, a paper and pencil test, required the child to connect a series of circles under time pressure. Trials included visual scanning, letter sequencing, number sequencing, and combined alternating letter‐number sequencing, and simple motor sequencing. *Color‐Word Interference* is a timed, four‐trial Stroop‐like task, requiring the child to name colors, read words, name the color of ink that does not match the word itself (inhibition), and alternately apply two rules during an inhibitory task depending on the presence of a cue (inhibition/switching). All D‐KEFS standardized scores have a mean of 10 and a standard deviation of 3, with higher scores indicating better performance.

Participants' parents completed the Child Behavior Checklist (CBCL) (Achenbach 2001) and a computerized version of the Kiddie Schedule for Affective Disorders and Schizophrenia (KSADS‐COMP) (Kaufman et al. 2021). CBCL standardized T‐scores have a mean of 50 and a standard deviation of 10, with higher scores indicating more behavioral abnormalities compared to same‐age peers.

## 3 Statistical Analyses

Statistical analyses were performed using IBM SPSS Statistics (Version 29.0.1.0). Prior to conducting parametric inferential statistics, the distributions of all continuous variables were assessed for normality. Shapiro–Wilk tests revealed deviations from normality with distributions showing slight negative skewness (as expected in a clinical population) for the SB‐5 VIQ (skewness = −0.733, SE = 0.319), FSIQ (skewness = −0.861, SE = 0.319), fluid reasoning (skewness = −0.529, SE = 0.319), and visual–spatial ability (skewness = −0.792, SE = 0.319) measures as well as for *D‐KEFS number‐letter sequencing* (skewness = −0.186, SE = 0.388). Given the modest deviations from normality, the sample size and the robustness of General Linear Models (GLMs) to non‐normality, the original scaled scores were retained for all primary analyses to facilitate more direct interpretation of the results.

Descriptive statistics were calculated to characterize the study sample. To evaluate potential attrition bias, baseline demographic and clinical characteristics were compared between participants who completed the follow‐up assessment and those lost to follow‐up. These were conducted using independent sample *t*‐tests for continuous variables and Pearson's chi‐square tests for categorical variables. As part of describing the sample, we present data from the KSADS‐COMP which characterize the participants in terms of DSM‐5 diagnoses. Data are presented by group (choline and placebo) merely as a check of the randomization and to show group equivalence. We had no specific hypotheses about choline and longitudinal diagnostic outcome.

Addressing the primary, a priori hypotheses, group differences in cognitive functioning at longitudinal follow‐up were examined with GLMs. Missing data were handled with listwise deletion. Differences in global cognitive functioning at follow‐up by treatment group (choline vs. placebo) were examined with two multivariate GLMs. The first GLM tested for treatment effect on the three overarching SB‐5 IQ scores (FSIQ, VIQ, and NVIQ) at follow‐up, controlling for baseline global cognitive functioning to account for pre‐existing individual differences (Mullen GCA or SB‐5 FSIQ, collected prior to study initiation), age at enrollment, and age at follow‐up. A second multivariate GLM tested for treatment effects on the five SB‐5 index scores (fluid reasoning, knowledge, quantitative reasoning, visual–spatial ability, and working memory), controlling for baseline global cognitive functioning (Mullen GCA or SB‐5 FSIQ), age at enrollment, and age at follow‐up. The potential impact of age at enrollment on cognitive outcome was evaluated by adding an interaction term (age at enrollment × treatment group) and re‐running the two GLMs described above. For secondary analyses, three selected subtests from the D‐KEFS administered at follow‐up were evaluated for treatment group differences (choline vs. placebo) with independent samples *t*‐tests. Similarly, three selected memory measures from the NEPSY‐II were evaluated with *t*‐tests. Because these measures were not administered at baseline, these tests were run without covariates using simple independent samples *t*‐tests with the follow‐up data. These were not corrected for multiple comparisons. For parent‐report behavioral measures (CBCL), two summary scores (Externalizing and Internalizing) were tested with GLMs that included baseline CBCL Internalizing and Externalizing measures as covariates. Lastly, choline dose–response relationships for cognitive measures that showed a significant choline effect were examined with linear regression analyses. Effect sizes are reported as partial eta squared and Cohen's *d* values (Cohen 1988), and the threshold for statistical significance was set at alpha = 0.05 (two‐tailed).

## 4 Results

### 4.1 Sample Descriptives

Relevant characteristics for the 58 participants who contributed data to the follow‐up are listed in Table 1. Participants were, on average, 3.9 years old at the time of enrollment in the initial RCT and 9.1 years old at the time of the longitudinal follow‐up study. FSIQ was 86.8 across all participants at baseline, reflecting a common finding of lower IQ in FASD (Coles et al. 1991; Mattson et al. 1997). As shown in Table 1, participants were well‐matched across groups (choline vs. placebo) for age, sex, baseline global cognitive functioning, ethnicity, race, and diagnosis. For descriptive purposes, all participants were characterized using updated, modified Institute of Medicine (IOM) diagnostic criteria (Hoyme et al. 2016), utilizing all available information from the child's medical records and from a dysmorphology evaluation conducted by the study physician (JKE) who has training and extensive experience with FASD evaluation. Of the 58 participants who contributed data to the longitudinal analyses, 48% met criteria for ARND, 43% met criteria for PFAS, and 9% met criteria for FAS. The proportion of participants with DSM‐5 diagnoses (past or present), as identified through the KSADS‐COMP parent‐interview, is listed in Table 2. In the full sample, high rates of externalizing disorders, including ADHD, Oppositional Defiant Disorder (ODD), and Conduct Disorder, were observed as expected from the literature (Khoury et al. 2018). High rates of anxiety disorders, including Separation Anxiety Disorder and Specific Phobia, were also observed.

**TABLE 1:** Demographic characteristics of enrolled participants who completed longitudinal follow‐up.

	Placebo (*n* = 30)	Choline (*n* = 28)	Statistical test
Age at enrollment [M(SD)]	3.86 (0.84)	3.87 (0.76)	*t*(56) = 0.011, *p* = 0.368
Age at follow‐up [M(SD)]	9.01 (1.30)	9.18 (1.26)	*t*(56) = 0.510, *p* = 0.581
Intelligence Quotient at enrollment [M(SD)]	86.73 (20.20)	86.81 (13.96)	*t*(54) = 0.016, *p* = 0.987
Intelligence Quotient at follow‐up [M(SD)]	86.93 (17.72)	89.88 (11.2)	*t*(54) = 0.731, *p* = 0.031*
Sex [*n*(%Female)]	17 (60.7%)	11 (39.3%)	*χ* ^2^ = 1.149, *p* = 0.284
Ethnicity [*n*(%Hispanic)]	4 (13.3%)	1 (3.6%)	*χ* ^2^ = 3.088, *p* = 0.378
Race^a^			*χ* ^2^ = 5.137, *p* = 0.399
[*n*(%American Indian/Alaska Native)]	4 (13.3%)	6 (21.4%)	—
[*n*(%Asian)]	2 (6.7%)	3 (10.7%)	—
[*n*(%Black or African American)]	8 (26.7%)	2 (7.1%)	—
[*n*(%White)]	11 (36.7%)	11 (39.3%)	—
[*n*(%Multiracial)]	5 (16.7%)	5 (17.8%)	—
[*n*(%Unknown)]	0 (0%)	1 (3.6%)	—
Diagnosis			*χ* ^*2*^ = 0.635, *p* = 0.728
Alcohol‐related neurodevelopmental disorder (ARND)	13 (43.3%)	15 (53.6%)	—
Partial fetal alcohol syndrome (pFAS)	14 (46.7%)	11 (39.3%)	—
Fetal alcohol syndrome (FAS)	3 (10.0%)	2 (7.1%)	—
Other drug exposure	15 (50.0%)	17 (60.7%)	*χ* ^2^ = 2.896, *p* = 0.235
History of global intellectual impairment	8 (26.7%)	5 (17.9%)	*χ* ^2^ = 0.646, *p* = 0.421
≥ 3 domains of impairment	29 (96.7%)	27 (96.4%)	*χ* ^2^ = 0.002, *p* = 0.960
Growth deficiency	6 (20.0%)	7 (25.0%)	*χ* ^2^ = 0.208, *p* = 0.648
Microcephaly	7 (23.3%)	7 (25.0%)	*χ* ^2^ = 0.052, *p* = 0.820
Dysmorphic face	17 (56.7%)	13 (46.4%)	*χ* ^2^ = 0.608, *p* = 0.436

^a^ Chi square tests reflect comparisons of each racial group to the proportion of participants who identified as White (e.g., proportion of participants who identified as Multiracial to proportion of those who identified as White); The diagnoses listed were made using updated, modified Institute of Medicine (IOM) criteria.

**TABLE 2:** DSM‐5 diagnoses (past or present) at follow‐up from Kiddie Schedule for Affective Disorders and Schizophrenia (KSADS).

Frequency	(*n*) % of the total sample (58)	(*n*) % of the placebo group (30)	(*n*) % of the choline group (28)	Chi‐square	Significance *p*
Separation anxiety disorder	(12) 20.7%	(5) 16.7%	(7) 25.0%	0.613	0.434
Attention‐deficit hyperactivity disorder (ADHD)	(11) 19.0%	(5) 16.7%	(6) 21.4%	0.214	0.644
Oppositional defiant disorder (ODD)	(11) 19.0%	(6) 20.0%	(5) 17.9%	0.043	0.835
Specific phobia	(10) 17.2%	(5) 16.7%	(5) 17.9%	0.014	0.950
Conduct disorder	(7) 12.1%	(4) 13.3%	(3) 10.7%	0.094	0.760
Suicidal ideation	(7) 12.1%	(6) 20.0%	(1) 3.6%	3.683	0.055
Autism	(4) 6.9%	(2) 6.7%	(2) 7.1%	0.005	0.943
Enuresis/Encopresis	(4) 6.9%	(3) 10.0%	(1) 3.6%	0.932	0.334
Social anxiety disorder	(3) 5.2%	(2) 6.7%	(1) 3.6%	0.283	0.595
Sleep problems	(3) 5.2%	(0) 0%	(3) 10.7%	3.390	0.066
Generalized anxiety disorder	(2) 3.4%	(2) 6.7%	(0) 0%	1.933	0.164
Mood disorder	(2) 3.4%	(1) 3.3%	(1) 3.6%	0.002	0.960
Obsessive–compulsive disorder	(2) 3.4%	(0) 0.0%	(2) 7.1%	2.219	0.136
Tic disorder	(2) 3.4%	(1) 3.3%	(1) 3.6%	0.002	0.969
Agoraphobia	(1) 1.7%	(0) 0%	(1) 3.6%	1.090	0.296
Post‐traumatic stress disorder	(1) 1.7%	(0) 0%	(1) 3.6%	1.090	0.296

### 4.2 Participants Seen at 5‐Year Follow‐Up Were Similar to Those Lost to Follow‐Up

Participants who received choline and those who received placebo were equally likely to return for follow‐up, chi‐square (1, 106) = 0.365, *p* = 0.546. There was not a significant difference for sex, but there was a trend for males to be slightly more likely to return for follow‐up, chi‐square (1, 117) = 3.082, *p* = 0.079. There was a significant difference for age, *t*(1, 111) = 2.021, *p* = 0.046, with the returning group having been enrolled at a slightly younger age (mean 3.87 years) than those lost to follow‐up (mean = 4.20 years). There was no difference for race, chi‐square (5, 117) = 7.046, *p* = 0.217, or ethnicity, chi‐square (1, 116) = 3.743, *p* = 0.291. Similarly, there was no difference in likelihood of returning by diagnosis, chi‐square (1, 109) = 1.564, *p* = 0.906, or other drug exposure, chi‐square (1, 111) = 2.986, *p* = 0.225. Lastly, the two groups did not have significantly different global cognitive functioning scores at baseline testing, *t*(1, 99) = 0.434, *p* = 0.666.

### 4.3 Participants Who Received Choline Scored Higher at Follow‐Up on Cognitive Index Scores Compared to Those Who Received Placebo

For the first GLM examining IQ scores, the omnibus test did not reach conventional levels of significance, Wilks lambda = 0.900, *F*(3, 47) = 1.747, *p* = 0.170, partial eta squared = 0.100, indicating that the overall multivariate profile of IQ scores did not differ significantly between groups. However, given the medium effect size (Cohen 1988), 10% of variance explained, the omnibus test's relative conservativeness, and the modest sample size, the follow‐up univariate tests were examined. Estimated Marginal Means (EMMs) and between group comparisons (choline vs. placebo) for the IQ scores are listed in Table 3. NVIQ scores were significantly higher for the choline group compared to placebo, *F*(1, 49) = 4.912, *p* = 0.031, with a medium effect size (Cohen's *d* = 0.611) and 9.1% of the variance explained by group. Participants who received choline had NVIQ scores that were 6.9% higher than those who had received placebo.

**TABLE 3:** Estimated marginal means and treatment group differences for Stanford Binet at longitudinal follow‐up.

EM Mean (SE)	Placebo	Choline	Statistic *F*	Significance *p*	Cohen's *d*
**Stanford Binet—5**					
Index scores					
Full‐scale IQ	86.7 (2.0)	91.6 (2.2)	2.701	0.107	0.452
Verbal IQ	88.3 (2.3)	91.6 (2.6)	0.900	0.347	0.261
Non‐verbal IQ	86.5 (1.9)	92.7 (2.1)	4.912	0.031*	0.611
Domain scores					
Fluid reasoning	92.2 (2.5)	92.6 (2.8)	0.013	0.909	0.031
Knowledge	83.4 (1.8)	87.9 (2.0)	2.653	0.110	0.448
Quantitative reasoning	91.4 (2.1)	94.3 (2.3)	0.887	0.351	0.259
Visual–spatial processing	92.7 (2.1)	97.8 (2.4)	2.578	0.115	0.442
Working memory	84.4 (2.2)	92.5 (2.4)	6.213	0.016*	0.687

*Note:* Results from Multivariate General Linear Models controlling for baseline cognitive functioning (Mullen Early Learning Composite or Stanford‐Binet Full‐Scale IQ), age at enrollment, and age at follow‐up.

* Significance *p* < 0.05.

For the second GLM examining cognitive index scores, the omnibus test did not reach conventional levels of significance, Wilks lambda = 0.796, *F*(5, 45) = 2.302, *p* = 0.060, partial eta squared = 0.204. However, there was a trend; the effect size was large, and 20.4% of the variance was explained by group (choline vs. placebo). Considering this, in the context of the modest sample size and the omnibus multivariate test's conservativeness, the follow‐up univariate tests were examined. EMMs and between groups comparisons for the index scores are listed in Table 3. The Working Memory Index was significantly higher for the choline group compared to placebo, *F*(1, 49) = 6.213, *p* = 0.016, with a medium effect size (Cohen's *d* = 0.687) and 11.3% of the variance explained. Participants who received choline had Working Memory Index scores that were more than half of a standard deviation (9.2%) higher than those who had received placebo.

For the three selected D‐KEFS executive functioning measures, no treatment group differences were seen for *Trail Making number‐letter sequencing*, *t*(35) = 0.656, *p* = 0.516, or *Color‐Word Interference inhibition‐switching*, *t*(34) = 1.596, *p* = 0.120. However, there was a non‐significant but medium‐effect size group difference for *Color‐Word Interference inhibition*, *t*(35) = 1.929, *p* = 0.062. Individuals who received choline had 30.2% higher scores (mean = 9.76, SD = 3.6) than those who had received placebo (mean = 7.20, SD = 4.4) with a Cohen's d effect size of 0.64.

For the three selected NEPSY‐II memory measures, no treatment group differences were seen for Memory for Faces delayed, *t*(52) = 0.453, *p* = 0.907, Memory for Names delayed, *t*(55) = 0.527, *p* = 0.600, or Narrative Memory free and cued total, *t*(55) = 0.393, *p* = 0.696.

### 4.4 Choline Was Not Associated With Parent‐Reported Behavior Change

For the CBCL, there was not a significant treatment group effect for Internalizing Problems, *F*(1, 55) = 0.736, *p* = 0.395, partial eta squared = 0.013, nor was there a significant treatment group effect for Externalizing Problems, *F*(1,55) = 0.085, *p* = 0.771, partial eta squared = 0.002.

### 4.5 Age at Enrollment

The interaction of age at enrollment and treatment group on overall cognitive function was non‐significant (SB‐5 IQ scores: Wilk's lambda = 0.934, *F*(3, 46) = 1.088, *p* = 0.364, partial eta squared = 0.066); SB‐5 index scores: Wilk's lambda = 0.925, *F*(5, 44) = 712, *p* = 0.687, partial eta squared = 0.075. Therefore, no further examination of age at enrollment as a potential moderator was pursued.

### 4.6 For Cognitive Domains Showing Improvement Following Choline, There Was a Significant Dose–Response Effect

Participants in the choline group were further evaluated to determine if choline dosage impacted cognitive functioning at follow‐up. Analyses were done only for SB‐5 NVIQ and Working Memory (which showed a significant choline effect) and D‐KEFS Color‐Word Interference inhibition (which showed a trend‐level effect). First, a linear regression analysis with SB‐5 NVIQ as the dependent measure and choline dose (range = 290.7–500.0 mg per day) and baseline global cognitive functioning as predictors was run. The overall model was significant: *R* = 0.557, *F*(2,23) = 4.728, *p* = 0.020 with dose explaining a significant amount of the variance in NVIQ, *B* = 0.061, SE = 0.022, *t*(23) = 2.764, *p* = 0.012. A second linear regression was performed with SB‐5 Working Memory Index as the dependent measure and choline dose and baseline global cognitive functioning as predictors. The overall model was significant: *R* = 0.579, *F*(2, 23) = 5.299, *p* = 0.014 and dose explained a significant amount of the variance in Working Memory Index, *B* = 0.062, *t*(23) = 2.320, *p* = 0.030. In each case, higher choline dose was associated with higher cognitive functioning at follow‐up, controlling for baseline global cognitive functioning. A third linear regression with D‐KEFS Color‐Word Interference Inhibition subtest score as the dependent measure and choline dose and baseline global cognitive functioning as predictors was run. The overall model was non‐significant: *R* = 0.152, *F*(2,14) = 0.143, *p* = 0.869.

## 5 Discussion

Here we report on a 5‐year longitudinal follow‐up study testing the durability of the cognitive effects of choline as a potential neurodevelopmental intervention in children with FASD. Compared to participants who received placebo, those treated with choline demonstrated significantly higher performance on tests of cognitive functioning including non‐verbal IQ and working memory, with medium to large effect sizes. Also, a non‐significant choline effect with a medium effect size was seen for one aspect of executive function (inhibition). There were no significant treatment group differences on measures of global cognitive functioning, memory, or parent‐rated behavior problems. We did not observe an effect of age on long‐term cognitive outcomes, but a higher dose of choline was associated with higher cognitive functioning at follow‐up.

Previously, we have shown beneficial effects of 9‐months of choline supplementation versus placebo in the domain of hippocampal‐dependent delayed sequential memory when measured immediately after study completion (Wozniak et al. 2025). Children who received choline performed 25% better on delayed recall than placebo. Age‐related improvements in delayed sequential memory were observed for those receiving choline, with younger participants (2.5 to < 4 years) showing greater improvement than older participants (4–5 years) (Wozniak et al. 2015). Because memory in this age range is foundational for all types of learning (Riggins et al. 2009), optimizing memory has important downstream implications for the child's future cognitive ability (Ramey and Ramey 1998). In a longitudinal study of typically developing children, delayed sequential memory performance at 20 months of age predicted up to 37% of the variance in explicit memory skill at 6 years of age (Riggins et al. 2013). To measure the downstream effects of optimizing memory functioning at a very early developmental stage, we brought back participants from three previous RCTs to evaluate choline's ability to alter long‐term neurodevelopmental trajectories in FASD.

The longitudinal data presented here revealed medium to large effects of choline on non‐verbal IQ, working memory, and one aspect of executive functioning. It is noteworthy that, when measured immediately after 9 months of supplementation, differences were not apparent for any of the global cognitive domains/IQ indices (Wozniak et al. 2015, 2025) but these benefits became evident after 5 years of additional development. In contrast, examining the memory measures included in the longitudinal study, we were not able to detect improvements that we hypothesized based on the initial improved performance on a hippocampus‐dependent form of memory. Thus, choline may impact cognitive functioning though a variety of mechanisms (not only hippocampal mechanisms) that may play out in unique ways over the course of long spans of child development.

Our findings remain consistent with previous studies demonstrating that choline's availability during important early developmental windows impacts numerous aspects of neurodevelopment. Nutritional supplementation during critical periods of brain development can broadly enhance neuroplasticity (Williams 2008). Improved neuroplasticity could, in theory, translate into improved learning and memory, executive functioning, and processing speed, among other domains (Rosso et al. 2025). Choline also plays a key role in the production of phosphatidylcholine, sphingomyelin, and plasmalogens present in all cell membranes. In neurons, these phospholipids are necessary for axonal growth and myelination, among other developmental processes (Zeisel and Niculescu 2006). In sum, existing preclinical studies suggest that the longitudinal findings of choline supplementation we present here are biologically plausible.

Preliminary evidence suggests that these mechanisms of choline may support brain development and function across a very long developmental period. In a separate longitudinal study of participants who returned 7 years after completing a choline trial (mean age 11.0 years), we measured a benefit of choline on lower‐order executive function skills (e.g., information processing speed) as well as a significant group difference in white matter organization (lower fiber dispersion) in the splenium of the corpus callosum (Gimbel et al. 2022). Notably, increased white matter organization in this region is correlated with better executive function performance. While preliminary and in need of further study, these results suggest choline‐related benefits for foundational memory functions may, in turn, support the later development of higher‐level cognitive functions in children with FASD. Together, choline may be contributing to the observed long‐term improvements in non‐verbal intelligence and working memory through a wide variety of these neurodevelopmental and experiential mechanisms or combinations, highlighting the need for a long‐term developmental perspective when evaluating outcomes from new treatments of this type.

In the current longitudinal outcome study, we did not observe an age interaction with choline, whereas we had previously reported that the effect size for the short‐term impact of choline on cognition is larger when given at earlier ages (Wozniak et al. 2015). To date, the only other published work examining choline supplementation for children with FASD, an RCT conducted by Nguyen and colleagues (Nguyen et al. 2016), examined older children (ages 5–10 years) and found no choline versus placebo differences after a much shorter (6 week) period of choline supplementation. Together, these findings suggest that there may be a window in early development for maximal benefit from choline supplementation (perhaps before age 4), with a protracted course of cognitive benefits that emerge over time. These data also suggest that longer periods of choline supplementation (ex. 9 months as in our studies) may yield benefits whereas shorter periods (6 weeks) may be inadequate. This specific question is currently being explored systematically in our 4th RCT (NCT05108974), which is testing 3‐months and 6‐months of choline supplementation against 9‐months and placebo (using the same weight‐based dosing as RCT3) in a design that will allow for direct comparison to our prior studies.

Appropriate dosing is another important factor to understand in potentially applying choline as a neurodevelopmental intervention. Here, using long‐term follow‐up data, we added to prior findings of dose–response relationships by showing that a higher choline dose (500 mg per day) was associated with better cognitive outcome (non‐verbal IQ and working memory) than a lower dose (290–500 mg based on 19 mg/kg of the child's weight). These findings with postnatal choline supplementation are generally consistent with studies of prenatal choline supplementation such as a study that found a relationship between maternal adherence (a proxy for dose received) and eyeblink conditioning performance as a measure of learning (Jacobson et al. 2018). In the same cohort, a dose–response relationship for choline was also observed for subcortical brain volumes including thalamus, caudate, putamen, and corpus callosum (Warton et al. 2021). In sum, the consistent finding of a dose–response relationship supports the RCT evidence that choline has a meaningful impact on cognition in FASD and highlights the importance of continuing to refine our understanding of dose, timing, etc.

This study has a number of limitations to acknowledge. First, the decision to conduct a longitudinal evaluation of the trial participants was made post hoc as opposed to being a planned component of the original studies. The 2‐year grant that funded the feasibility work in RCT1 and the 3‐year grant that funded RCT2 covered only the trials themselves. Funds supporting RC3 then allowed for the initiation of the longitudinal follow‐up study. This sequence of events contributed to lower participation in the longitudinal study (the early participants were not told to expect a follow‐up study, nor were they tracked intensively in anticipation of one). As a result, a significant number were lost to follow‐up. In addition, once it became apparent that longitudinal follow‐up would yield critical information about early choline supplementation, a decision was made for RCT3 to drop the Mullen Scales in favor of the SB‐5 which can be administered across a much wider age range. For the current longitudinal analysis, maximizing the power was important with the small available sample and, so, data were combined. Although effect sizes for the change in cognitive functioning following choline versus placebo were derived from the RCTs (Cohen's *d* = 0.66), the longitudinal follow‐up was not based on an additional power analysis; instead, the sample presented here is composed of all available participants who were willing to participate. In sum, the statistical power of this longitudinal study that evolved in stages is lower than it would be in an idealized, a priori, large‐scale longitudinal trial covering a 10–15‐year span and, therefore, some effects may have been missed or underestimated by the current analyses. Lastly, the study's evolution resulted in two different baseline measures of global cognitive functioning being used over the three RCTs. Although the Mullen and SB‐5 are not completely equivalent, both batteries measure a range of cognitive/developmental skills, both provide an index of global cognitive functioning that is related to general intelligence or “g factor”, and both are sensitive to neurodevelopmental delays (Bishop et al. 2011; Farmer et al. 2016; Mullen 1995; Swineford et al. 2015). Nonetheless, it is important to acknowledge the limitations resulting from the inclusion of different baseline measures of cognitive functioning in the current analyses.

At this point, choline is the only biological, evidence‐based intervention that has been systematically tested in FASD. As a potential treatment for the neurodevelopmental aspects of FASD, choline has a number of favorable qualities including acceptability, tolerability, convenience, low risk, and low cost. We have measured high rates of acceptance for choline by parents of children with FASD, and we have demonstrated that oral choline in a liquid form is not any more difficult to take than placebo, even for periods as long as 9 months (Wozniak et al. 2013). The only adverse effect that we have measured in all of our trials has been fishy body odor, which is dose‐related and manageable by dose adjustments or dose‐splitting. Across all of our pilot work and RCTs of choline supplementation in 164 children with FASD (Wozniak et al. 2025) with more than 41,000 doses of choline (or placebo) administered, the data support its safety and efficacy and highlight the power of early neurodevelopmental interventions for individuals impacted by PAE.

## 6 Conclusions

Early nutritional supplementation with choline, which directly impacts brain development and cognitive functioning, represents a potentially powerful, scalable, cost‐effective intervention for young children with FASD. Perhaps more importantly, this longitudinal study and the initial RCTs that came before serve as a proof of principle that early developmental intervention has the potential to alter the neurodevelopmental trajectory of the individual and to improve cognitive functioning over the long term.

## Funding

This work was supported by the National Institutes of Health (NIH) grants R21AA019580, R33AA019580, R01AA024123, and R56AA024123 (Wozniak & Georgieff).

## Conflicts of Interest

The authors declare no conflicts of interest.

## Data Availability

The data that support the findings of this study are available from the corresponding author upon reasonable request.

## References

[acer70394-bib-0001] Achenbach, T. M. 2001. Manual for the Child Behavior Checklist/6–18. University of Vermont, Department of Psychiatry.

[acer70394-bib-0002] Achenbach, T. M. , and L. A. Rescorla . 2000. Manual Fo ASEBA Preschool Forms and Profiles. University of Vermont, Research Center for Children, Youth, and Families.

[acer70394-bib-0003] Akison, L. K. , J. Kuo , N. Reid , R. N. Boyd , and K. M. Moritz . 2018. “Effect of Choline Supplementation on Neurological, Cognitive, and Behavioral Outcomes in Offspring Arising From Alcohol Exposure During Development: A Quantitative Systematic Review of Clinical and Preclinical Studies.” Alcoholism, Clinical and Experimental Research 42, no. 9: 1591–1611. 10.1111/acer.13817 29928762

[acer70394-bib-0004] Albright, C. D. , C. B. Friedrich , E. C. Brown , M. H. Mar , and S. H. Zeisel . 1999. “Maternal Dietary Choline Availability Alters Mitosis, Apoptosis and the Localization of TOAD‐64 Protein in the Developing Fetal Rat Septum.” Brain Research. Developmental Brain Research 115, no. 2: 123–129. 10.1016/s0165-3806(99)00057-7 10407130

[acer70394-bib-0005] Albright, C. D. , A. Y. Tsai , C. B. Friedrich , M. H. Mar , and S. H. Zeisel . 1999. “Choline Availability Alters Embryonic Development of the Hippocampus and Septum in the Rat.” Brain Research. Developmental Brain Research 113, no. 1–2: 13–20. 10.1016/s0165-3806(98)00183-7 10064869

[acer70394-bib-0006] Bekdash, R. A. 2019. “Neuroprotective Effects of Choline and Other Methyl Donors.” Nutrients 11, no. 12: 2995. 10.3390/nu11122995 PMC6950346 31817768

[acer70394-bib-0007] Bishop, S. L. , W. Guthrie , M. Coffing , and C. Lord . 2011. “Convergent Validity of the Mullen Scales of Early Learning and the Differential Ability Scales in Children With Autism Spectrum Disorders.” American Journal on Intellectual and Developmental Disabilities 116, no. 5: 331–343. 10.1352/1944-7558-116.5.331 PMC7398154 21905802

[acer70394-bib-0008] Castaldelli‐Maia, J. M. , L. E. Segura , and S. S. Martins . 2021. “The Concerning Increasing Trend of Alcohol Beverage Sales in the U.S. During the COVID‐19 Pandemic.” Alcohol 96: 37–42. 10.1016/j.alcohol.2021.06.004 PMC8421038 34245809

[acer70394-bib-0009] Chasnoff, I. J. , A. M. Wells , and L. King . 2015. “Misdiagnosis and Missed Diagnoses in Foster and Adopted Children With Prenatal Alcohol Exposure.” Pediatrics 135, no. 2: 264–270. 10.1542/peds.2014-2171 25583914

[acer70394-bib-0010] Cheng, R.‐K. , C. J. MacDonald , C. L. Williams , and W. H. Meck . 2008. “Prenatal Choline Supplementation Alters the Timing, Emotion, and Memory Performance (TEMP) of Adult Male and Female Rats as Indexed by Differential Reinforcement of Low‐Rate Schedule Behavior.” Learning & Memory 15, no. 3: 153–162. 10.1101/lm.729408 PMC2275657 18323570

[acer70394-bib-0011] Cohen, J. 1988. Statistical Power Analysis for the Behavioral Sciences. L Erlbaum Associates.

[acer70394-bib-0012] Coles, C. D. , R. T. Brown , I. E. Smith , K. A. Platzman , S. Erickson , and A. Falek . 1991. “Effects of Prenatal Alcohol Exposure at School Age. I. Physical and Cognitive Development.” Neurotoxicology and Teratology 13, no. 4: 357–367. 10.1016/0892-0362(91)90084-a 1921915

[acer70394-bib-0013] Coles, C. D. , J. A. Kable , C. L. Keen , et al. 2015. “Dose and Timing of Prenatal Alcohol Exposure and Maternal Nutritional Supplements: Developmental Effects on 6‐Month‐Old Infants.” Maternal and Child Health Journal 19, no. 12: 2605–2614. 10.1007/s10995-015-1779-x PMC4644455 26164422

[acer70394-bib-0014] Conlay, L. A. , and S. H. Zeisel . 1982. “Neurotransmitter Precursors and Brain Function.” Neurosurgery 10, no. 4: 524–529. 10.1227/00006123-198204000-00021 6124895

[acer70394-bib-0015] Delis, D. C. , E. Kaplan , and J. H. Kramer . 2001. Delis‐Kaplan Executive Function System (D‐KEFS). Harcourt Assessment, Inc.

[acer70394-bib-0016] Ernst, A. M. , B. A. Gimbel , E. de Water , et al. 2022. “Prenatal and Postnatal Choline Supplementation in Fetal Alcohol Spectrum Disorder.” Nutrients 14: 3. 10.3390/nu14030688. PMC8837993 35277047

[acer70394-bib-0017] Farmer, C. , C. Golden , and A. Thurm . 2016. “Concurrent Validity of the Differential Ability Scales, Second Edition With the Mullen Scales of Early Learning in Young Children With and Without Neurodevelopmental Disorders.” Child Neuropsychology: A Journal on Normal and Abnormal Development in Childhood and Adolescence 22, no. 5: 556–569. 10.1080/09297049.2015.1020775 PMC5588686 25833070

[acer70394-bib-0018] Flannigan, K. , K. D. Coons‐Harding , T. Anderson , et al. 2020. “A Systematic Review of Interventions to Improve Mental Health and Substance Use Outcomes for Individuals With Prenatal Alcohol Exposure and Fetal Alcohol Spectrum Disorder.” Alcoholism, Clinical and Experimental Research 44, no. 12: 2401–2430. 10.1111/acer.14490 PMC7839542 33119894

[acer70394-bib-0019] Gimbel, B. A. , M. E. Anthony , A. M. Ernst , et al. 2022. “Long‐Term Follow‐Up of a Randomized Controlled Trial of Choline for Neurodevelopment in Fetal Alcohol Spectrum Disorder: Corpus Callosum White Matter Microstructure and Neurocognitive Outcomes.” Journal of Neurodevelopmental Disorders 14, no. 1: 59. 10.1186/s11689-022-09470-w PMC9756672 36526961

[acer70394-bib-0020] Glenn, M. J. , E. D. Kirby , E. M. Gibson , et al. 2008. “Age‐Related Declines in Exploratory Behavior and Markers of Hippocampal Plasticity Are Attenuated by Prenatal Choline Supplementation in Rats.” Brain Research 1237: 110–123. 10.1016/j.brainres.2008.08.049 PMC2677022 18786518

[acer70394-bib-0021] Hoyme, H. E. , W. O. Kalberg , A. J. Elliott , et al. 2016. “Updated Clinical Guidelines for Diagnosing Fetal Alcohol Spectrum Disorders.” Pediatrics 138, no. 2: 4256. 10.1542/peds.2015-4256. PMC4960726 27464676

[acer70394-bib-0022] Idrus, N. M. , K. R. Breit , and J. D. Thomas . 2017. “Dietary Choline Levels Modify the Effects of Prenatal Alcohol Exposure in Rats.” Neurotoxicology and Teratology 59: 43–52. 10.1016/j.ntt.2016.11.007 PMC5770193 27888055

[acer70394-bib-0023] Jacobson, S. W. , R. C. Carter , C. D. Molteno , et al. 2018. “Efficacy of Maternal Choline Supplementation During Pregnancy in Mitigating Adverse Effects of Prenatal Alcohol Exposure on Growth and Cognitive Function: A Randomized, Double‐Blind, Placebo‐Controlled Clinical Trial.” Alcoholism, Clinical and Experimental Research 42, no. 7: 1327–1341. 10.1111/acer.13769 PMC6028282 29750367

[acer70394-bib-0024] Kable, J. A. , C. D. Coles , C. L. Keen , et al. 2015. “The Impact of Micronutrient Supplementation in Alcohol‐Exposed Pregnancies on Information Processing Skills in Ukrainian Infants.” Alcohol 49, no. 7: 647–656. 10.1016/j.alcohol.2015.08.005 PMC4636447 26493109

[acer70394-bib-0025] Kaufman, J. , K. Kobak , B. Birmaher , and N. de Lacy . 2021. “KSADS‐COMP Perspectives on Child Psychiatric Diagnostic Assessment and Treatment Planning.” Journal of the American Academy of Child and Adolescent Psychiatry 60, no. 5: 540–542. 10.1016/j.jaac.2020.08.470 33385508

[acer70394-bib-0026] Khoury, J. E. , B. Jamieson , and K. Milligan . 2018. “Risk for Childhood Internalizing and Externalizing Behavior Problems in the Context of Prenatal Alcohol Exposure: A Meta‐Analysis and Comprehensive Examination of Moderators.” Alcoholism, Clinical and Experimental Research 42, no. 8: 1358–1377. 10.1111/acer.13805 29852057

[acer70394-bib-0027] Korkman, M. , U. Kirk , and S. Kemp . 2007. NEPSY‐II: Second Edition. PsychCorp.

[acer70394-bib-0028] Lange, S. , C. Probst , G. Gmel , J. Rehm , L. Burd , and S. Popova . 2017. “Global Prevalence of Fetal Alcohol Spectrum Disorder Among Children and Youth: A Systematic Review and Meta‐Analysis.” JAMA Pediatrics 171, no. 10: 948–956. 10.1001/jamapediatrics.2017.1919 PMC5710622 28828483

[acer70394-bib-0029] Li, Q. , S. Guo‐Ross , D. V. Lewis , et al. 2004. “Dietary Prenatal Choline Supplementation Alters Postnatal Hippocampal Structure and Function.” Journal of Neurophysiology 91, no. 4: 1545–1555. 10.1152/jn.00785.2003 14645379

[acer70394-bib-0030] Mattson, S. N. , E. P. Riley , L. Gramling , D. C. Delis , and K. L. Jones . 1997. “Heavy Prenatal Alcohol Exposure With or Without Physical Features of Fetal Alcohol Syndrome Leads to IQ Deficits.” Journal of Pediatrics 131, no. 5: 718–721. 10.1016/s0022-3476(97)70099-4 9403652

[acer70394-bib-0031] May, P. A. , C. D. Chambers , W. O. Kalberg , et al. 2018. “Prevalence of Fetal Alcohol Spectrum Disorders in 4 US Communities.” JAMA: The Journal of the American Medical Association 319, no. 5: 474–482. 10.1001/jama.2017.21896 PMC5839298 29411031

[acer70394-bib-0032] Meck, W. H. , and C. L. Williams . 1997. “Characterization of the Facilitative Effects of Perinatal Choline Supplementation on Timing and Temporal Memory.” Neuroreport 8, no. 13: 2831–2835. 10.1097/00001756-199709080-00005 9376513

[acer70394-bib-0033] Meck, W. H. , and C. L. Williams . 2003. “Metabolic Imprinting of Choline by Its Availability During Gestation: Implications for Memory and Attentional Processing Across the Lifespan.” Neuroscience and Biobehavioral Reviews 27, no. 4: 385–399. 10.1016/s0149-7634(03)00069-1 12946691

[acer70394-bib-0034] Moore, E. M. , and E. P. Riley . 2015. “What Happens When Children With Fetal Alcohol Spectrum Disorders Become Adults?” Current Developmental Disorders Reports 2, no. 3: 219–227. 10.1007/s40474-015-0053-7 PMC4629517 26543794

[acer70394-bib-0035] Mullen, E. M. 1995. Mullen Scales of Early Learning. AGS.

[acer70394-bib-0036] Nguyen, T. T. , R. D. Risbud , S. N. Mattson , C. D. Chambers , and J. D. Thomas . 2016. “Randomized, Double‐Blind, Placebo‐Controlled Clinical Trial of Choline Supplementation in School‐Aged Children With Fetal Alcohol Spectrum Disorders.” American Journal of Clinical Nutrition 104, no. 6: 1683–1692. 10.3945/ajcn.116.142075 PMC5118735 27806977

[acer70394-bib-0037] O'Connor, M. J. , B. Shah , S. Whaley , P. Cronin , B. Gunderson , and J. Graham . 2002. “Psychiatric Illness in a Clinical Sample of Children With Prenatal Alcohol Exposure.” American Journal of Drug and Alcohol Abuse 28, no. 4: 743–754. 10.1081/ada-120015880 12492268

[acer70394-bib-0038] Panczakiewicz, A. L. , L. Glass , C. D. Coles , et al. 2016. “Neurobehavioral Deficits Consistent Across Age and Sex in Youth With Prenatal Alcohol Exposure.” Alcoholism, Clinical and Experimental Research 40, no. 9: 1971–1981. 10.1111/acer.13153 PMC5008991 27430360

[acer70394-bib-0039] Popova, S. , D. Dozet , M.‐R. Faulkner , L. Howie , and V. Temple . 2024. “Prenatal Exposures, Diagnostic Outcomes, and Life Experiences of Children and Youths With Fetal Alcohol Spectrum Disorder.” Nutrients 16, no. 11: 1655. 10.3390/nu16111655 PMC11174948 38892588

[acer70394-bib-0040] Pyapali, G. K. , D. A. Turner , C. L. Williams , W. H. Meck , and H. S. Swartzwelder . 1998. “Prenatal Dietary Choline Supplementation Decreases the Threshold for Induction of Long‐Term Potentiation in Young Adult Rats.” Journal of Neurophysiology 79, no. 4: 1790–1796. 10.1152/jn.1998.79.4.1790 9535948

[acer70394-bib-0041] Ramey, C. T. , and S. L. Ramey . 1998. “Early Intervention and Early Experience.” American Psychologist 53, no. 2: 109–120. 10.1037//0003-066x.53.2.109 9491742

[acer70394-bib-0042] Riggins, T. , N. C. Miller , P. J. Bauer , M. K. Georgieff , and C. A. Nelson . 2009. “Electrophysiological Indices of Memory for Temporal Order in Early Childhood: Implications for the Development of Recollection.” Developmental Science 12, no. 2: 209–219. 10.1111/j.1467-7687.2008.00757.x PMC2771175 19143795

[acer70394-bib-0043] Riggins, T. , C. L. Cheatham , E. Stark , and P. J. Bauer . 2013. “Elicited Imitation Performance at 20 Months Predicts Memory Abilities in School‐Aged Children.” Journal of Cognition and Development: Official Journal of the Cognitive Development Society 14, no. 4: 593–606. 10.1080/15248372.2012.689392 PMC3891774 24436638

[acer70394-bib-0044] Roid, G. H. 2003. Stanford Binet Intelligence Scales. Fifth ed. Riverside.

[acer70394-bib-0045] Rosso, C. , P. R. Brustio , J. Manuello , and A. Rainoldi . 2025. “Neuroplasticity of Brain Networks Through Exercise: A Narrative Review About Effect of Types, Intensities, and Durations.” Sports 13, no. 8: 280. 10.3390/sports13080280 PMC12390317 40863789

[acer70394-bib-0046] Sarkar, D. K. , O. Gangisetty , J. R. Wozniak , et al. 2019. “Persistent Changes in Stress‐Regulatory Genes in Pregnant Women or Children Exposed Prenatally to Alcohol.” Alcoholism, Clinical and Experimental Research 43, no. 9: 1887–1897. 10.1111/acer.14148 PMC6722014 31329297

[acer70394-bib-0047] Sawant, O. B. , S. M. Birch , C. R. Goodlett , T. A. Cudd , and S. E. Washburn . 2019. “Maternal Choline Supplementation Mitigates Alcohol‐Induced Fetal Cranio‐Facial Abnormalities Detected Using an Ultrasonographic Examination in a Sheep Model.” Alcohol 81: 31–38. 10.1016/j.alcohol.2019.05.001 PMC6842443 31082506

[acer70394-bib-0048] Swineford, L. B. , W. Guthrie , and A. Thurm . 2015. “Convergent and Divergent Validity of the Mullen Scales of Early Learning in Young Children With and Without Autism Spectrum Disorder.” Psychological Assessment 27, no. 4: 1364–1378. 10.1037/pas0000116 PMC11107881 25894712

[acer70394-bib-0049] Thomas, J. D. , J. S. Biane , K. A. O'Bryan , T. M. O'Neill , and H. D. Dominguez . 2007. “Choline Supplementation Following Third‐Trimester‐Equivalent Alcohol Exposure Attenuates Behavioral Alterations in Rats.” Behavioral Neuroscience 121, no. 1: 120–130. 10.1037/0735-7044.121.1.120 17324056

[acer70394-bib-0050] Waddell, J. , and S. M. Mooney . 2017. “Choline and Working Memory Training Improve Cognitive Deficits Caused by Prenatal Exposure to Ethanol.” Nutrients 9, no. 10: 1080. 10.3390/nu9101080 PMC5691697 28961168

[acer70394-bib-0051] Warton, F. L. , C. D. Molteno , C. M. R. Warton , et al. 2021. “Maternal Choline Supplementation Mitigates Alcohol Exposure Effects on Neonatal Brain Volumes.” Alcoholism, Clinical and Experimental Research 45, no. 9: 1762–1774. 10.1111/acer.14672 PMC8526390 34342017

[acer70394-bib-0052] Williams, C. L. 2008. “Food for Thought: Brain, Genes, and Nutrition.” Brain Research 1237: 1–4. 10.1016/j.brainres.2008.09.039 18928791

[acer70394-bib-0053] Wozniak, J. R. , A. J. Fuglestad , J. K. Eckerle , et al. 2013. “Choline Supplementation in Children With Fetal Alcohol Spectrum Disorders Has High Feasibility and Tolerability.” Nutrition Research 33, no. 11: 897–904. 10.1016/j.nutres.2013.08.005 PMC3815698 24176229

[acer70394-bib-0054] Wozniak, J. R. , A. J. Fuglestad , J. K. Eckerle , et al. 2015. “Choline Supplementation in Children With Fetal Alcohol Spectrum Disorders: A Randomized, Double‐Blind, Placebo‐Controlled Trial.” American Journal of Clinical Nutrition 102, no. 5: 1113–1125. 10.3945/ajcn.114.099168 PMC4625582 26447156

[acer70394-bib-0055] Wozniak, J. R. , E. P. Riley , and M. E. Charness . 2019. “Clinical Presentation, Diagnosis, and Management of Fetal Alcohol Spectrum Disorder.” Lancet Neurology 18, no. 8: 760–770. 10.1016/S1474-4422(19)30150-4 PMC6995665 31160204

[acer70394-bib-0056] Wozniak, J. R. , B. A. Fink , A. J. Fuglestad , et al. 2020. “Four‐Year Follow‐Up of a Randomized Controlled Trial of Choline for Neurodevelopment in Fetal Alcohol Spectrum Disorder.” Journal of Neurodevelopmental Disorders 12, no. 1: 9. 10.1186/s11689-020-09312-7 PMC7066854 32164522

[acer70394-bib-0057] Wozniak, J. R. , J. K. Eckerle , B. A. Gimbel , et al. 2025. “Choline Enhances Elicited Imitation Memory Performance in Preschool Children With Prenatal Alcohol Exposure: A Cumulative Report of 3 Randomized Controlled Trials.” American Journal of Clinical Nutrition 121, no. 4: 921–931. 10.1016/j.ajcnut.2025.02.009 PMC12002193 39956364

[acer70394-bib-0058] Zeisel, S. H. 2006. “The Fetal Origins of Memory: The Role of Dietary Choline in Optimal Brain Development.” Journal of Pediatrics 149, no. 5 Suppl: S131–S136. 10.1016/j.jpeds.2006.06.065 PMC2430654 17212955

[acer70394-bib-0059] Zeisel, S. H. 2011. “The Supply of Choline Is Important for Fetal Progenitor Cells.” Seminars in Cell & Developmental Biology 22, no. 6: 624–628. 10.1016/j.semcdb.2011.06.002 PMC3188336 21693194

[acer70394-bib-0060] Zeisel, S. H. , and M. D. Niculescu . 2006. “Perinatal Choline Influences Brain Structure and Function.” Nutrition Reviews 64, no. 4: 197–203. 10.1111/j.1753-4887.2006.tb00202.x PMC2438605 16673755

